# Textile‐Integrated Electrochemical Sensing Approaches for Monitoring Wound‐Relevant Biochemical Markers

**DOI:** 10.1111/iwj.71083

**Published:** 2026-10-29

**Authors:** Adnan Maroof Khan, Johannes Wendler, Yvonne Zimmermann, Dilbar Aibibu, Michael Wöltje, Chokri Cherif

**Affiliations:** ^1^ Institute of Textile Machinery and High Performance Material Technology, Technische Universität Dresden Dresden Germany; ^2^ Institut für Fördertechnik und Kunststoffe, Technische Universität Chemnitz Chemnitz Germany; ^3^ Textilforschungsinstitut Thueringen‐Vogtland e.V.—TITV Greiz, Yvonne Zimmermann Greiz Germany

**Keywords:** biocompatibility, enzymatic lactate sensing, hydrogen peroxide sensing, textile electrochemical sensors, textile‐integrated electrochemical biosensors, wound dressing applications

## Abstract

Wearable electrochemical sensors offer promising opportunities for wound‐related biochemical assessment by enabling the detection of wound‐relevant biomarkers directly at the skin–wound interface. In this study, we present a textile‐integrated sensor platform for dual‐analyte response of hydrogen peroxide (H_2_O_2_) and enzymatically quantified lactate, which are key indicators of oxidative stress and metabolic activity, respectively. The redox sensor was fabricated using platinum‐coated Shieldex working electrodes and Ag/AgCl reference electrodes, arranged in a coaxial winding yarn. Besides having the same type of reference electrode in the setup, the lactate sensor employed a gold‐based working electrode functionalized with a chitosan–PPD–lactate oxidase (LOx) multilayer along with a platinum‐based counter electrode. Electrochemical characterisation showed that the redox sensor exhibited consistent current–time response behaviour, approaching a quasi‐stabilised response level within approximately 180 s, and demonstrating a measurable response trend across a broad experimentally explored H_2_O_2_ concentration range (from the micromolar to high millimolar range). The lactate sensor produced repeatable qualitative current increases of approximately 25% and 50% at 2 mM and 4 mM lactate, respectively, with response magnitude affected by pH and temperature. Biocompatibility testing confirmed no cytotoxic effects for gold‐ and platinum‐based electrodes, and only moderate effects for Ag/AgCl under prolonged incubation, supporting the suitability of the primary sensing elements for wound‐contact applications. Taken together, the results demonstrate proof‐of‐concept functionality under controlled laboratory conditions, while comprehensive analytical validation remains the subject of future work, which supports the potential of textile‐based electrochemical sensors for integration into next‐generation smart wound dressings. Future research will also focus on environmental compensation, enzyme stabilisation and further system optimisation for wound‐relevant operation.

## Key Points

- Textile‐integrated electrochemical sensors were developed for proof‐of‐concept monitoring of wound‐relevant hydrogen peroxide and lactate.
- A coaxially wound redox sensor demonstrated a measurable response to hydrogen peroxide under controlled laboratory conditions.
- A braided, enzyme‐functionalized lactate sensor showed repeatable qualitative current increases at 2 mM and 4 mM lactate.
- In vitro cytocompatibility testing supported the suitability of gold‐ and platinum‐based sensing elements for further wound‐dressing development, while comprehensive analytical validation remains necessary.

## 1 Introduction

Chronic and infected wounds remain a major healthcare challenge because they are frequently associated with delayed healing, persistent inflammation, biofilm formation and multidrug‐resistant (MDR) bacterial infections, which reduce the effectiveness of conventional antimicrobial therapies and increase treatment costs. Consequently, considerable research has focused on advanced wound management strategies that combine therapeutic intervention with continuous monitoring of the wound microenvironment. Emerging approaches, including ozone‐based antimicrobial therapy, oxygen delivery platforms and microneedle‐assisted antibacterial systems, have demonstrated considerable potential for improving wound healing while supporting more targeted wound management and reducing dependence on systemic antibiotics [1, 2, 3, 4, 5].

The integration of electrochemical sensing technologies into textile substrates has emerged as a transformative direction in wearable healthcare diagnostics. Particularly in wound assessment and care‐related applications, smart textiles can enable the non‐invasive, near real‐time indication of biochemical markers, offering clinicians earlier insight into complications such as infection, chronic inflammation or tissue degradation [6, 7]. Among the most wound‐relevant biomarkers for wound assessment and qualitative condition evaluation are hydrogen peroxide (H_2_O_2_), a reactive oxygen species linked to oxidative stress processes, and lactate, a metabolic byproduct reflective of cellular respiration and tissue perfusion status [8, 9, 10]. Previous studies, including work by Ma et al., have shown that textile‐based sensing platforms can respond to such analytes under mechanically demanding and humid conditions, highlighting their suitability for wearable and wound‐contact use scenarios [11].

The conformability, wearability and integration of sensing elements into everyday materials may be restricted by the use of rigid electrodes or flexible films applied to the skin in traditional sensor systems [12, 13]. In contrast, textile‐integrated sensors offer high breathability, softness and wear comfort while maintaining direct skin contact, making them particularly suitable for wound dressing applications [14, 15]. Advances in textile electrode engineering now include multi‐layer architectures, protective membranes and nanostructured coatings to enhance both functional response characteristics and operational stability under physiological conditions [16, 17]. Alongside these therapeutic developments, significant progress has also been made in wound‐monitoring technologies aimed at providing continuous assessment of the wound microenvironment. Flexible electrochemical sensors, wearable pH sensing systems, oxygen delivery‐monitoring platforms and smart closed‐loop wound dressings have demonstrated the ability to monitor wound status and, in some cases, provide responsive therapeutic intervention [5, 18, 19]. These advances illustrate the growing importance of integrating sensing functionality directly into wound dressings. However, many reported systems rely on flexible films or rigid electronic components rather than textile‐engineered sensor architectures that are compatible with established textile manufacturing processes.

Textile yarn‐based electrochemical sensors with winding or braided structures are particularly attractive in this context because they provide mechanical robustness, structural uniformity and compatibility with scalable textile manufacturing processes [20, 21]. Consistent geometries and protective encapsulation of active elements are made possible by braiding techniques, which also maintain electrical connectivity under repeated mechanical deformation [20]. Similar multi‐electrode textile systems have demonstrated simultaneous qualitative response behaviour to more than one biomarker in biofluids, indicating that integrated wound‐related sensing is feasible [22]. Recent developments in H_2_O_2_‐responsive textile or flexible sensors, for instance, have employed nanostructured platinum interfaces integrated into flexible supports to achieve improved response behaviour under physiological conditions [23].

Lactate remains a key biochemical marker in both medical and sports contexts, and several recent works have explored wearable lactate sensors using enzymatic recognition layers combined with conductive textile substrates [24]. In particular, the coupling of lactate oxidase (LOx) with conductive polymers or carbon‐based nanomaterials has been shown to improve signal consistency and reduce environmental influences [17]. These strategies are directly relevant to wound care, where local pH and temperature variations can influence biochemical sensor responses.

Both sensor types described in this study were developed using exclusively textile‐compatible materials and scalable fabrication processes. This work addresses a research gap in the development of mechanically robust textile electrode architectures for wound‐relevant biochemical sensing, using hydrogen peroxide and lactate as model analytes representing oxidative stress and metabolic activity, respectively. Unlike many previously reported textile electrochemical biosensors that primarily focus on individual sensing elements or electrode materials, the present study emphasises textile‐engineered sensor architectures designed for practical integration into wound dressing systems. The proposed coaxial winding redox sensor and triple‐helix braided lactate sensor combine textile‐compatible fabrication, mechanical flexibility and electrochemical functionality within yarn‐based sensing platforms. Accordingly, the present study focuses on establishing the feasibility of textile‐integrated electrochemical sensing rather than providing a fully analytically validated biosensing system. The work therefore demonstrates the feasibility of an architecture‐driven textile sensing platform for wound‐relevant biochemical monitoring rather than the development of a single analytically optimised biosensor. The following sections describe the fabrication, characterisation and performance evaluation of the textile sensor yarns, with emphasis on their structural design, functional behaviour and response trends under environmentally relevant conditions.

## 2 Materials and Methods

### 2.1 Materials

Commercially available materials were used for sensor fabrication and electrochemical testing under controlled laboratory conditions relevant to wound‐sensing applications. Shieldex silver‐plated polyamide monofilament yarn (diameter 100 μm; Statex Produktions‐ und Vertriebs GmbH, Bremen, Germany) was used as the conductive base for electrode preparation and further processed by TITV e.V. (Greiz, Germany). Chitosan (medium molecular weight, 95% degree of deacetylation; Heppe Medical Chitosan GmbH, Halle (Saale), Germany) and poly(p‐phenylenediamine) (≥ 98% purity; Sigma‐Aldrich Chemie GmbH, Steinheim, Germany) were used in the enzymatic sensing layer formulation for lactate‐responsive electrodes. Lactate oxidase (LOx, ≥ 20 U/mg, from *Aerococcus viridans* ; Toyobo Co. Ltd., Osaka, Japan) was used as the biocatalyst for lactate sensing. Analytical grade phosphate‐buffered saline (PBS, pH 7.4; Merck KGaA, Darmstadt, Germany) served as the electrolyte medium for all electrochemical measurements to approximate physiological conditions.

Metal wires used included platinum wire (99.99% purity; Goodfellow GmbH, Hamburg, Germany), gold wire (99.99% purity; Goodfellow GmbH, Hamburg, Germany) and silver wire (99.9% purity; Goodfellow GmbH, Hamburg, Germany). Additional reagents comprised glutaraldehyde solution (25%; Sigma‐Aldrich Chemie GmbH, Steinheim, Germany), sulphuric acid (H_2_SO_4_, ≥ 95%; Merck KGaA, Darmstadt, Germany), hydrogen peroxide (H_2_O_2_, 30%; Merck KGaA, Darmstadt, Germany) and acetic acid (≥ 99%; Merck KGaA, Darmstadt, Germany), all of analytical grade. All chemicals were used without further purification.

### 2.2 Textile Sensor Fabrication

#### 2.2.1 Redox Sensor

The redox sensor was constructed using a conductive textile yarn platform tailored for electrochemical sensing under wound‐relevant conditions. The working electrode (WE) was prepared from Shieldex silver‐plated polyamide monofilament yarn and subsequently coated with platinum under controlled electroplating parameters (0.5–0.75 A/dm^2^, 480 s) to achieve a uniform and continuous catalytic surface. This coating process provided adequate catalytic activity and corrosion resistance, and reproducible coating quality across multiple samples. The viscose yarn (2 × 16 tex ≈32 tex per strand, arranged in multiple plies) used as a structural component in the yarn construction was produced in‐house on an Airjet J20 air spinning machine (Rieter GmbH, Switzerland) and twisted on a DirecTwist laboratory twisting machine (AGTEKS, Turkey) to maintain consistent dimensions and mechanical stability.

The reference electrode (RE) was fabricated by electrochemically chloridating silver‐coated yarn in sodium chloride solution immediately after silver deposition, producing an Ag/AgCl surface. For the sensor yarn assembly, the Ag/AgCl yarn was first closely wound with an insulating material to define a controlled distance from the Pt‐coated yarn. The Pt‐coated filament and the insulated Ag/AgCl yarn were then guided in parallel and further wound with another insulating thread, so that both electrodes were held together within a single yarn. This coaxial winding structure provides mechanical robustness, maintains consistent electrode spacing and retains sufficient flexibility for textile integration. Figure 1b shows representative implementation variants of the redox winding structure yarn. A two‐electrode configuration was employed for redox sensing experiments, with the WE and RE serving as the active pair in PBS solution.

**FIGURE 1:**
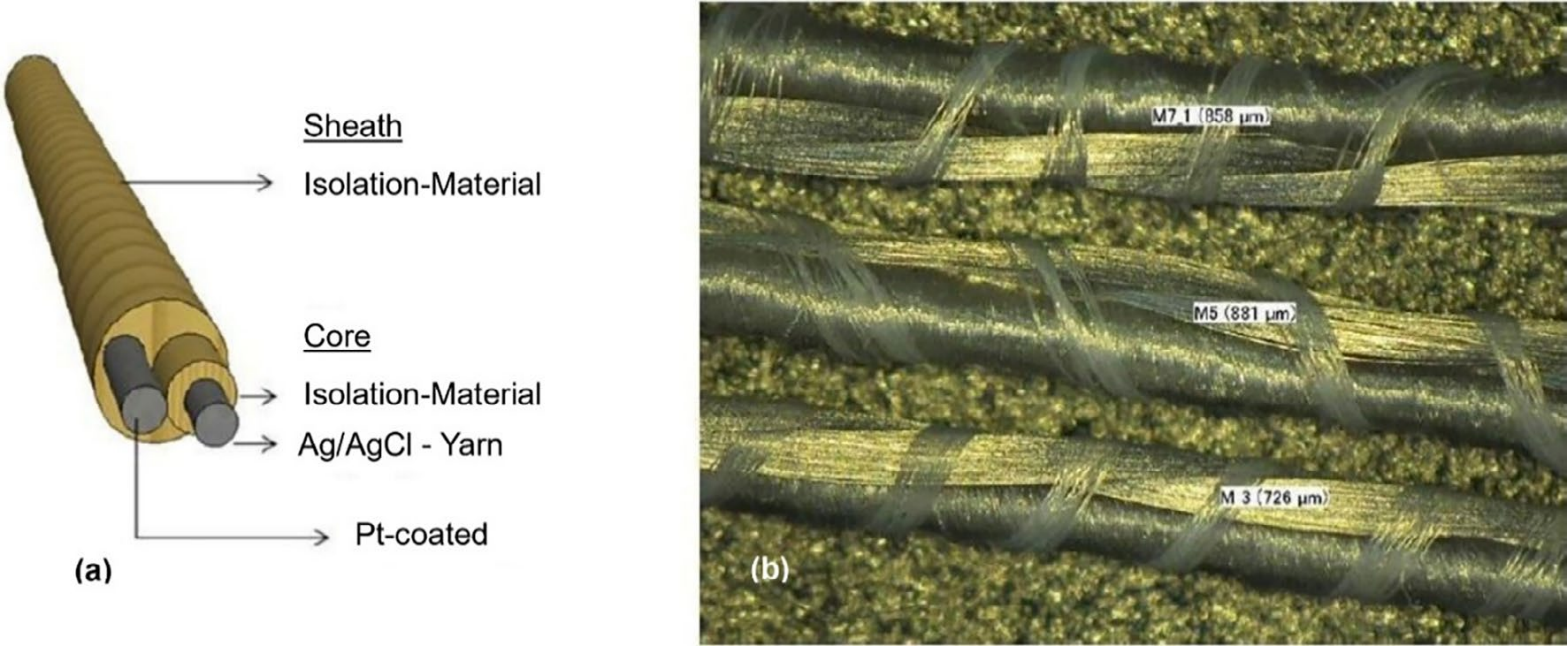
Structure of the coaxial winding structure of the redox sensor yarn: (a) schematic diagram of the electrode arrangement within the winding structure; (b) various implementation variants of the winding structure.

The morphology and elemental composition of the platinum‐coated conductive yarn were later evaluated by optical and SEM/EDX imaging, as summarised in Figure 9.

#### 2.2.2 Lactate Sensor

The lactate sensor design integrated an enzymatic multilayer sensing concept with a mechanically robust braided electrode architecture. The sensing principle (Figure 2) relied on the enzymatic transformation of lactate into pyruvate and hydrogen peroxide by lactate oxidase (LOx), while the electrochemical signal was recorded at the working electrode via the electrochemical response associated with the generated hydrogen peroxide. The electrode surface was functionalized with a permselective poly(p‐phenylenediamine) (PPD) membrane to allow hydrogen peroxide diffusion while excluding larger interferents, a chitosan hydrogel layer to provide an enzyme‐compatible environment, and immobilised LOx for qualitative lactate‐responsive signal generation.

**FIGURE 2:**
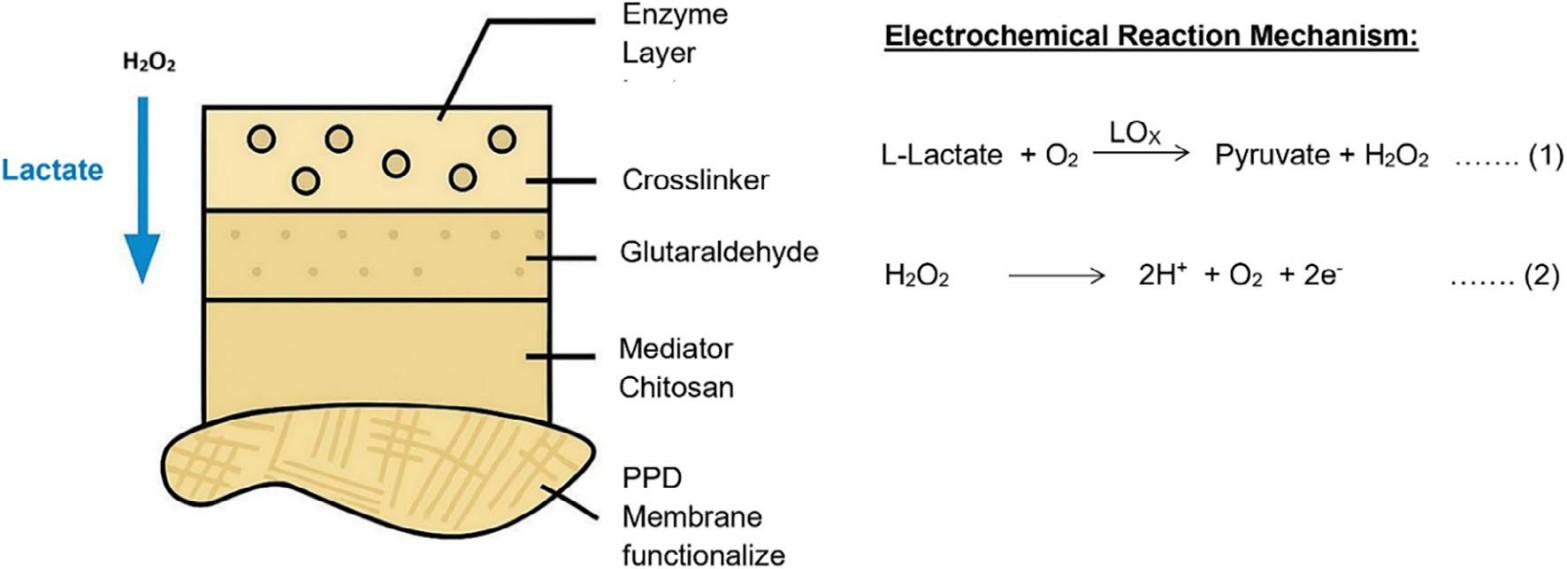
Conceptual illustration of the enzymatic sensing mechanism for lactate, showing electrode layer configuration and associated electrochemical reaction steps.

For mechanical stability and textile integration, the electrodes were arranged in a triple‐helix braid (Figure 3a–c) consisting of a gold‐based working electrode functionalized with a chitosan–PPD–lactate oxidase (LOx) multilayer, an Ag/AgCl‐coated silver reference electrode and a platinum counter electrode. The platinum counter electrode was included to provide a current‐balancing sink, allowing the potential of the gold working electrode to be controlled versus the Ag/AgCl reference during lactate sensing experiments. The in‐house produced viscose yarn (2 × 16 tex) was included as an insulating and structural component to maintain electrode spacing, prevent electrical shorting and enhance flexibility. The braid was produced on an August Herzog RU 2/12–80 braiding machine (August Herzog Maschinenfabrik GmbH & Co. KG, Oldenburg, Germany) using a 1‐over‐1 triple‐helix braiding pattern with bobbin type ASDh 80 in a double‐pull configuration. This architecture enabled stable electrode positioning and mechanical integrity suitable for textile‐based wound‐contact applications, as illustrated in Figure 3.

**FIGURE 3:**
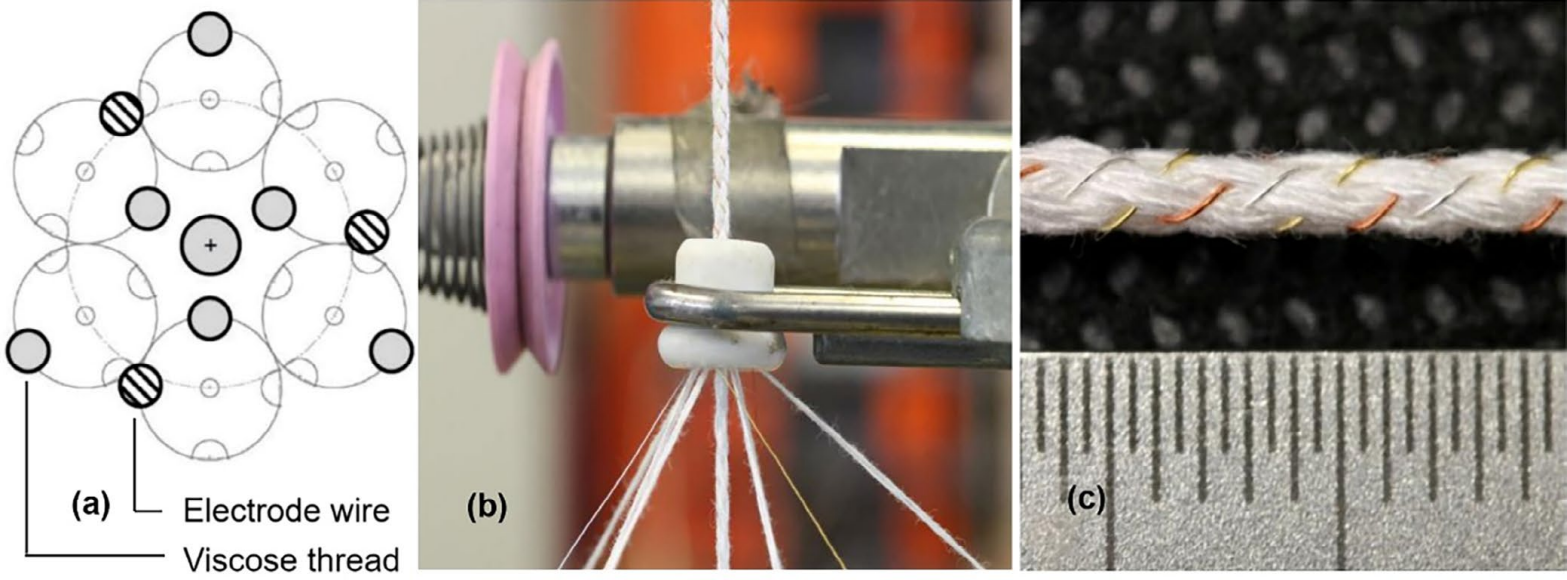
Triple‐helix lactate sensor structure: (a) schematic illustration of the braided electrode arrangement, (b, c) photographs of the braiding process using electrode wires and viscose support yarn.

### 2.3 Enzyme Immobilisation for Lactate Detection

The working electrode was coated with a functional multilayer designed to enable lactate‐responsive electrochemical signal generation. Following surface cleaning with piranha solution (H_2_SO_4_:H_2_O_2_, 7:3), a permselective poly(p‐phenylenediamine) (PPD) membrane was formed via electropolymerisation at 0.9 V versus Ag/AgCl in a 50 mM PPD phosphate‐buffered solution over 20 h. This membrane permits hydrogen peroxide diffusion while blocking larger interferents. A chitosan hydrogel layer (3% w/v, degree of deacetylation 95%, dissolved in 3% acetic acid) was then deposited using alternating dips in chitosan and 25% glutaraldehyde to form a cross‐linked, enzyme‐compatible matrix. The visual progression of these coating steps is shown in Figure 4.

**FIGURE 4:**
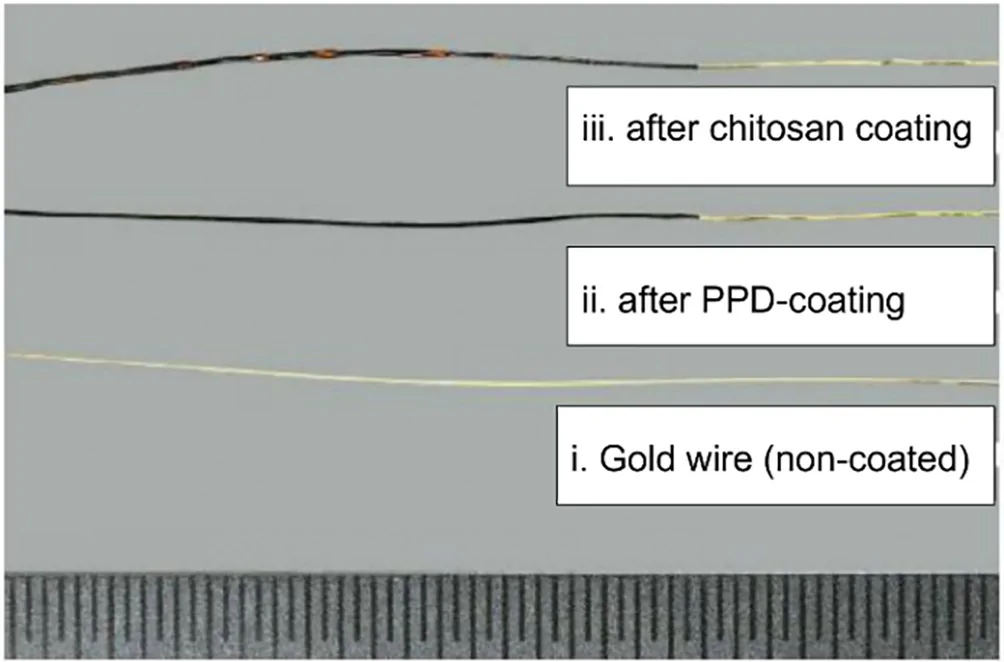
Sequential coating process for the lactate sensor working electrode: (i) uncoated gold wire, (ii) PPD‐coated wire, (iii) chitosan‐coated wire.

The coated electrodes were rinsed in PBS to remove excess glutaraldehyde. Lactate oxidase (LOx) (20 U in 100 μL PBS) was applied by drop‐casting onto the chitosan layer, with covalent immobilisation achieved via Schiff base formation between aldehyde and amine groups. The prepared sensors were dried and stored in PBS at 4°C prior to sensing experiments. This multilayer strategy supported stable enzyme immobilisation and reproducible lactate‐responsive signal trends under the tested laboratory conditions.

### 2.4 Electrochemical Testing

All electrochemical measurements were carried out in phosphate‐buffered saline (PBS, pH 7.4) at room temperature (25°C ± 1°C) using a portable potentiostat (EmStat3+, PalmSens). A two‐ or three‐electrode configuration was selected depending on the sensor type. The working electrode was either a platinum‐coated conductive filament (for redox sensors) or a noble metal wire with enzymatic modification (for lactate sensors). The reference electrode was an Ag/AgCl‐coated silver yarn, while the counter electrode (used only for lactate measurements) was a platinum wire.

#### 2.4.1 Redox Sensor Testing

The redox sensor yarn was designed to exhibit H_2_O_2_‐related current response behaviour under wound‐relevant conditions, reflecting oxidative stress‐associated activity. The sensor was operated under constant‐potential amperometric conditions vs. Ag/AgCl in phosphate‐buffered saline (PBS, pH 7.4). Sensor responses were recorded at a fixed response time of 180 s, following the addition of the analyte, using two operating potentials (+12 and + 30 mV).

Hydrogen peroxide was added in a sequential dilution series from 1 μM to 0.1 M, and the corresponding qualitative concentration‐dependent current response trends were recorded. The selected response time of 180 s was used to ensure temporally stabilised signal readout and improved comparability across concentration levels, rather than to establish analytical steady‐state conditions.

#### 2.4.2 Lactate Sensor Testing

Lactate sensing experiments were performed in a three‐electrode arrangement at a fixed potential of +300 mV versus Ag/AgCl. Lactate concentrations (0, 2 and 4 mM) were added sequentially, with additional tests conducted under varying pH conditions (6.0, 7.4 and 8.0) and elevated temperature (40°C) to assess environmental influences on the sensor response behaviour.

All tests were conducted in repeated runs (*n* = 3) to confirm consistency of the observed sensing behaviour, and data were processed using PSTrace software and Microsoft Excel. These measurements were intended to demonstrate functional feasibility and application‐oriented response behaviour of the textile‐integrated sensors, rather than to establish full analytical performance metrics such as calibration curves, detection limits or clinical validation.

## 3 Results

### 3.1 Redox Sensor Performance Evaluation

#### 3.1.1 Time‐Dependent Amperometric Response

Hydrogen peroxide sensing behaviour was examined under the amperometric conditions as described earlier, using the selected applied potential of +12 mV versus Ag/AgCl. As shown in Figure 5, the response of the redox sensor in 12.8 mM hydrogen peroxide exhibits an initial current change during the first ~20 s, followed by a gradual, time‐dependent signal evolution towards more negative current values over the recorded measurement period. After approximately 180 s, the current evolution becomes less pronounced, indicating a quasi‐stabilised response level suitable for comparative evaluation across measurement conditions.

**FIGURE 5:**
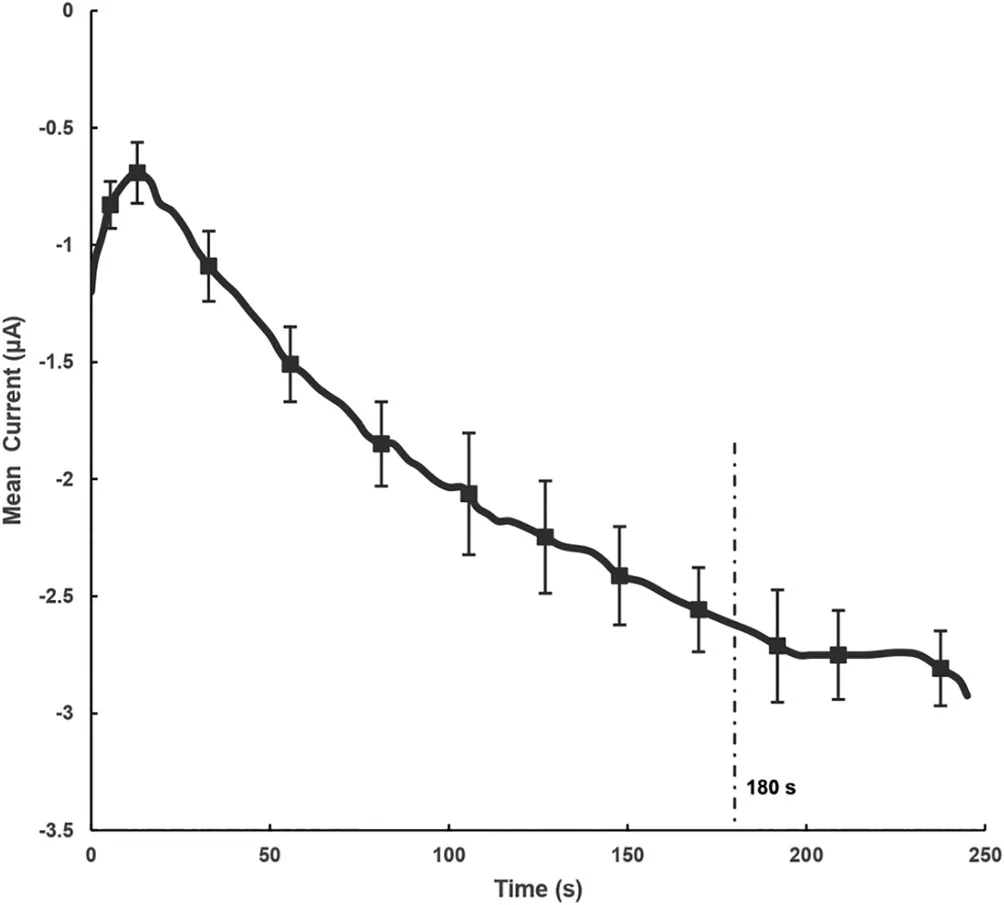
Time‐dependent amperometric response of the coaxial winding redox sensor measured in 12.8 mM hydrogen peroxide at +12 mV versus Ag/AgCl.

Such behaviour is consistent with slow equilibration processes at the textile‐based electrode interface, including wetting of the fibres, diffusion through the winding structure and gradual access of H_2_O_2_ to the Pt surface. This response characteristic is typical for porous, fibre‐based electrodes, where electrolyte penetration and diffusion pathways develop progressively during operation.

The current continued to evolve over time and did not reach a strict steady‐state plateau within the observed time window, which is expected for textile‐integrated electrochemical systems. Repeated measurements showed a similar response trend with modest variation, indicating consistent directional behaviour across runs of the textile electrode structure.

These observations indicate that the redox yarn provides a measurable and reproducible current–time signal evolution under wound‐relevant H_2_O_2_ conditions. On this basis, a response time of 180 s was selected as a practical and reproducible reference point for subsequent concentration‐dependent performance comparisons.

#### 3.1.2 Dose–Response to H_2_O_2_ Concentration

The concentration‐dependent behaviour of the redox sensor was evaluated by sequential additions of hydrogen peroxide under constant‐potential amperometric conditions described earlier. Figure 6 shows the amperometric response (recorded at +12 mV vs. Ag/AgCl after 180 s) plotted against the logarithmic concentration range from approximately 10 μmol/L to 0.1 mol/L.

**FIGURE 6:**
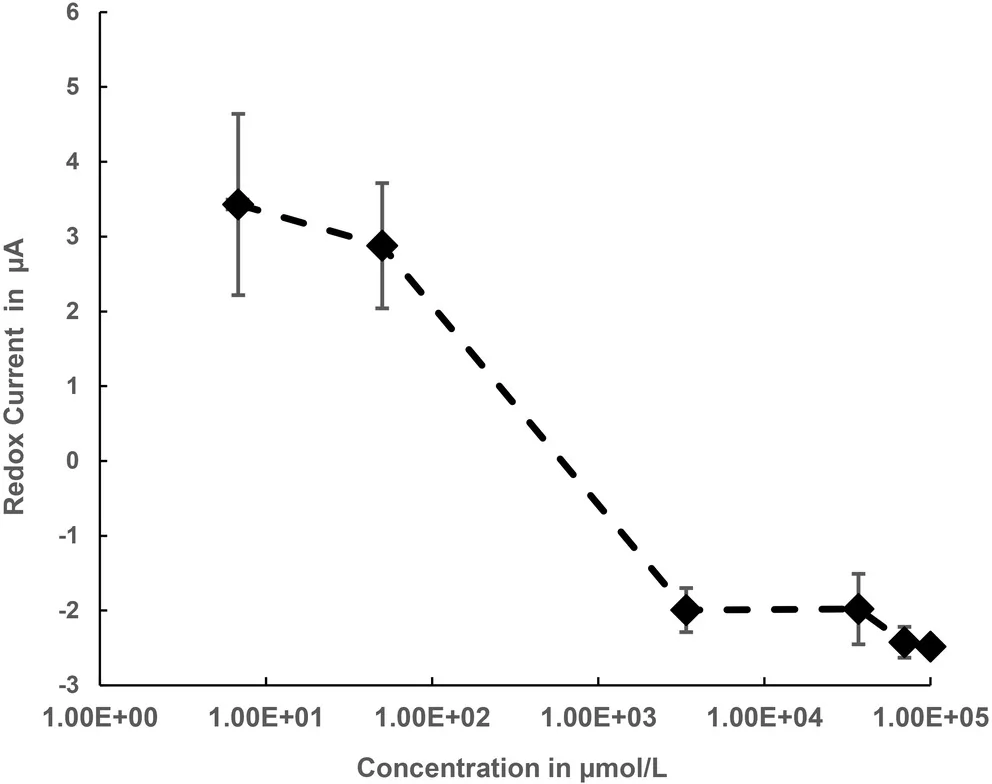
Concentration‐dependent amperometric response trend of the coaxial winding redox sensor to hydrogen peroxide (H_2_O_2_) at +12 mV versus Ag/AgCl, evaluated at a fixed response time of 180 s.

Across the tested range, the sensor exhibited a clear decrease in current with increasing H_2_O_2_ concentration, following a systematic concentration‐dependent response trend. This behaviour is consistent with diffusion‐related access of hydrogen peroxide to the platinum‐coated surface within the textile winding structure. At lower concentrations, the current changed more steeply, while at higher concentrations the curve gradually approached a flatter region, reflecting limitations associated with mass transport and surface interactions in fibre‐based electrodes.

The measurement series showed a similar directional response trend across repeated runs, reflecting consistent concentration‐dependent behaviour of the textile redox yarn under wound‐relevant conditions. Although the fibre‐based structure does not produce an ideal steady‐state plateau or analytically calibrated response, the observed concentration‐dependent signal evolution provides a measurable and interpretable response trend, suitable for proof‐of‐concept and semi‐quantitative assessment of oxidative stress‐related markers in wound environments (Table 1).

**TABLE 1:** Redox sensor architecture and operational characteristics.

Parameter	Observation/value
Sensor architecture	Coaxial winding textile yarn
Working electrode	Pt‐coated Shieldex conductive yarn
Reference electrode	Ag/AgCl‐coated silver yarn
Operating potential	+12 mV (selected operating condition)
Electrolyte	PBS (pH 7.4)
Response time	~180 s (quasi‐stabilised response)
H_2_O_2_ concentration range tested	~1 μM to high mM range (experimentally explored)
Signal behaviour	Qualitative, concentration‐dependent current trend
Reproducibility	Consistent directional response across repeated runs
Limitations	Signal variability influenced by diffusion and wetting effects inherent to textile‐based architectures

### 3.2 Lactate Sensor Performance

#### 3.2.1 Electrochemical Response to Lactate Concentrations

Lactate solutions with concentrations of 2 mM and 4 mM, as well as phosphate‐buffered saline (PBS) as the reference, were used to assess the electrochemical performance of the lactate sensor. As shown in Figure 7, the current–time curves exhibit a concentration‐dependent increase in current response behaviour upon the addition of lactate. The PBS baseline remained largely unchanged throughout the measurement, while 2 mM lactate induced an average relative current increase of approximately 25%, and 4 mM lactate produced an increase of around 50%, relative to the PBS baseline under identical measurement conditions.

**FIGURE 7:**
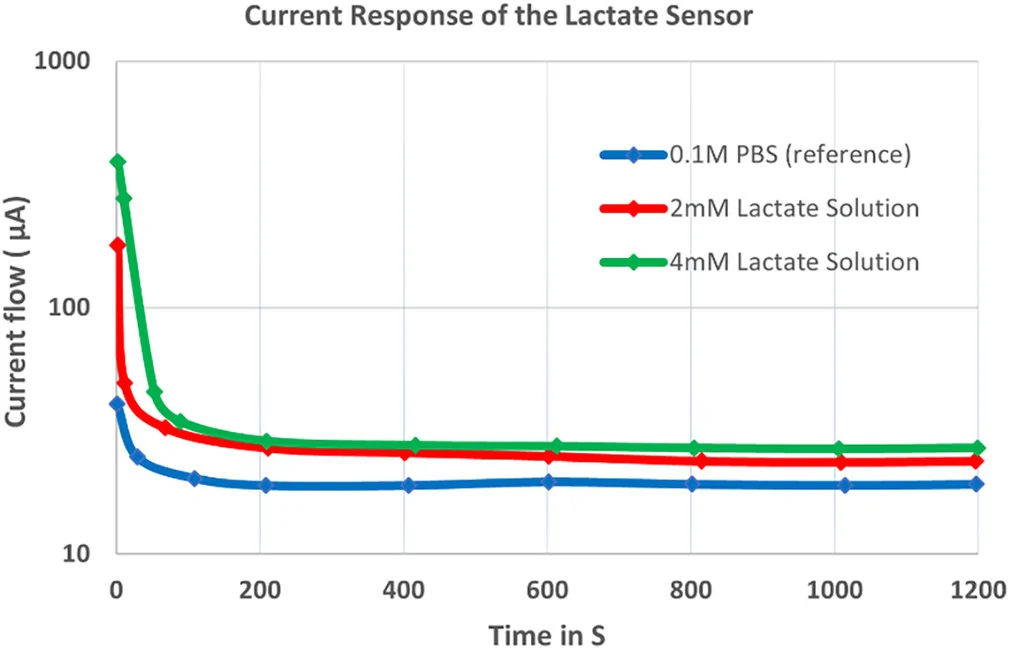
Current–time response of the lactate sensor in 0.1 M PBS (blue), 2 mM lactate (red) and 4 mM lactate (green) solutions.

The initial transient current rise observed after lactate addition is attributed to the enzymatic activity of lactate oxidase (LOx), which converts lactate into hydrogen peroxide (H_2_O_2_), followed by gradual signal stabilisation influenced by diffusion and enzyme–electrode interactions. These results indicate a clear and reproducible qualitative response trend to physiologically relevant lactate concentration levels, supporting the functional feasibility of the textile‐integrated enzymatic sensing concept.

#### 3.2.2 Mechanical Stability Under Bending

Mechanical robustness was evaluated by measuring the capacitance of the sensors before and after repeated bending cycles, intended to qualitatively simulate mechanical deformation expected in wearable wound dressing applications. As shown in Figure 8, the absolute capacitance values and the corresponding relative capacitance change (%) were recorded for the lactate sensor yarn, textile‐integrated sensor and non‐textile‐integrated sensor configurations.

**FIGURE 8:**
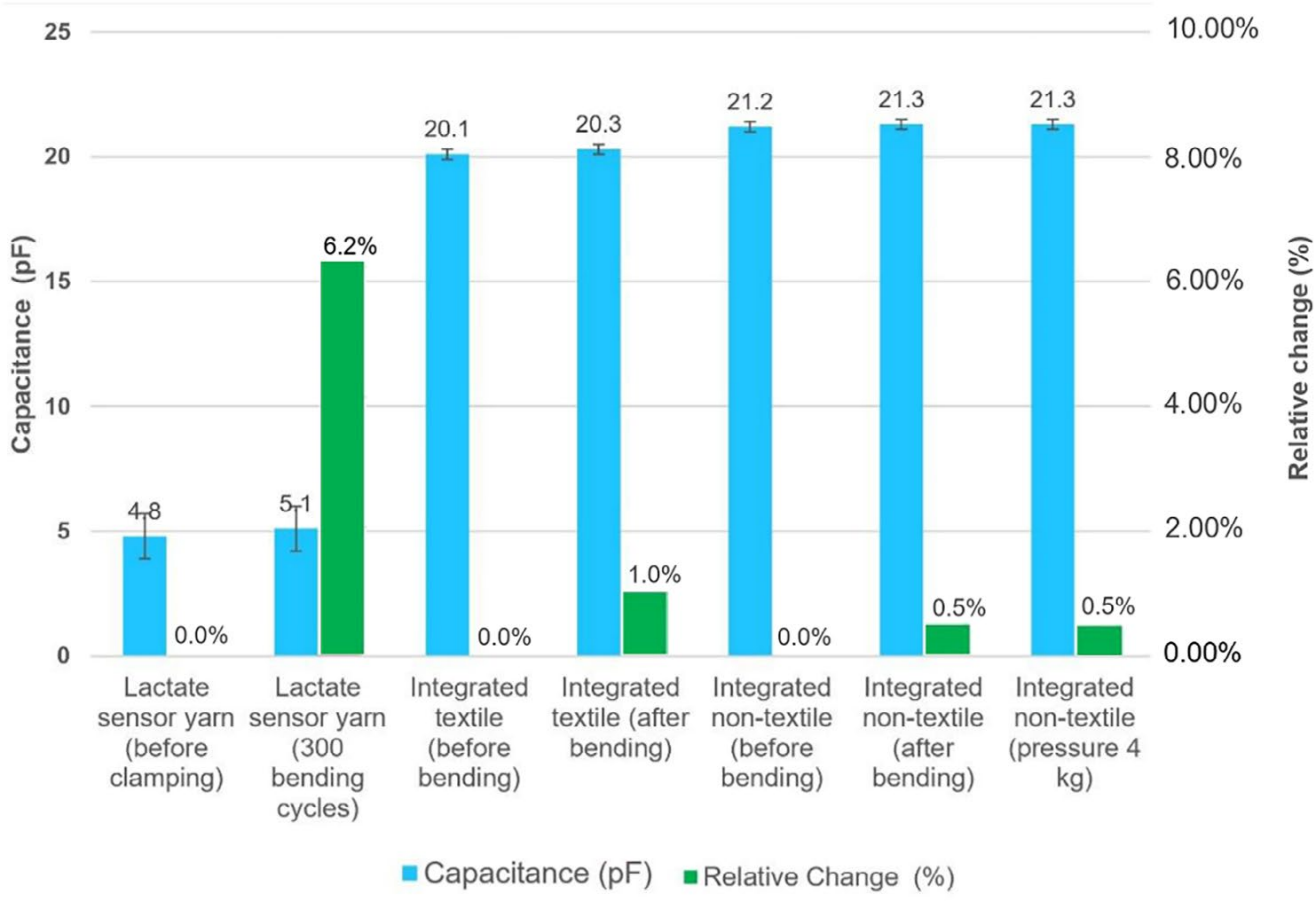
Capacitance values (pF, blue bars) and relative capacitance changes (%; green bars) of the lactate sensor yarn, textile‐integrated sensor and non‐textile‐integrated sensor before and after 300 bending cycles and under static applied pressure (4 kg).

Across all sensor formats, capacitance showed no pronounced or systematic change after 300 bending cycles, indicating that the overall structural integrity and electrical continuity of the sensor elements were largely preserved. The lactate sensor yarn exhibited a maximum relative change of approximately 6% after bending, whereas both the textile‐integrated and non‐textile‐integrated configurations showed only minor changes (≤ 1%).

These variations indicate that the mechanical stress introduced during repeated bending had a limited influence on the electrical characteristics of the sensors. The results suggest that the braided architecture, together with the selected electrode and structural yarn materials, provides a robust and flexible configuration suitable for repeated deformation. The ability to retain stable electrical behaviour after mechanical loading represents an important functional requirement for wearable wound‐related applications, where sensors are exposed to frequent movement and handling.

#### 3.2.3 Environmental Sensitivity to pH and Temperature

Selectivity tests were performed to evaluate the sensor's response to environmental parameters such as pH variation and temperature change (Table 2). These tests were intended to assess qualitative robustness under physiologically relevant wound conditions rather than full analytical selectivity. In comparison to neutral conditions, the current slightly decreased at pH 6.0, while a modest increase was observed at pH 8.0. Elevated temperature (40°C) resulted in a small increase in current, which is consistent with enhanced enzyme activity at higher temperatures.

**TABLE 2:** Influence of pH and temperature on the qualitative lactate sensor response.

Condition	Observed effect on current	Interpretation
Neutral PBS	Baseline	Reference condition
2 mM lactate	~+ 25% current increase	Positive response due to enzymatic reaction
4 mM lactate	~+ 50% current increase	Strong enzymatic signal
Temperature 40°C	Slight increase	Enzyme kinetics increase
pH 6.0	Moderate suppression	Reduced enzyme activity
pH 8.0	Slight enhancement	Mild increase in enzyme reactivity

Importantly, the magnitude of these variations was substantially smaller than the lactate‐induced signal, indicating that the sensor response is primarily governed by enzymatic lactate conversion rather than by moderate environmental fluctuations. These results suggest that the textile‐based lactate sensor maintains functional stability under pH and temperature ranges relevant to wound environments.

### 3.3 Microscopic and SEM Characterisation of Electrodes

Surface morphology of the sensor electrodes was assessed using optical microscopy and scanning electron microscopy (SEM), supplemented with energy‐dispersive X‐ray spectroscopy (EDX) where applicable, to qualitatively evaluate coating uniformity, structural integrity and fabrication reproducibility. For the reference electrode, a silver substrate was electrochemically chloridated to form an Ag/AgCl layer. As shown in Figure 9a–d, optical microscopy (500×) and SEM imaging indicated the successful formation of the AgCl coating. The optical images illustrate a clear change in surface texture and reflectivity after chloridation, while SEM analysis revealed a rough, granular morphology consistent with AgCl layer formation reported in the literature.

**FIGURE 9:**
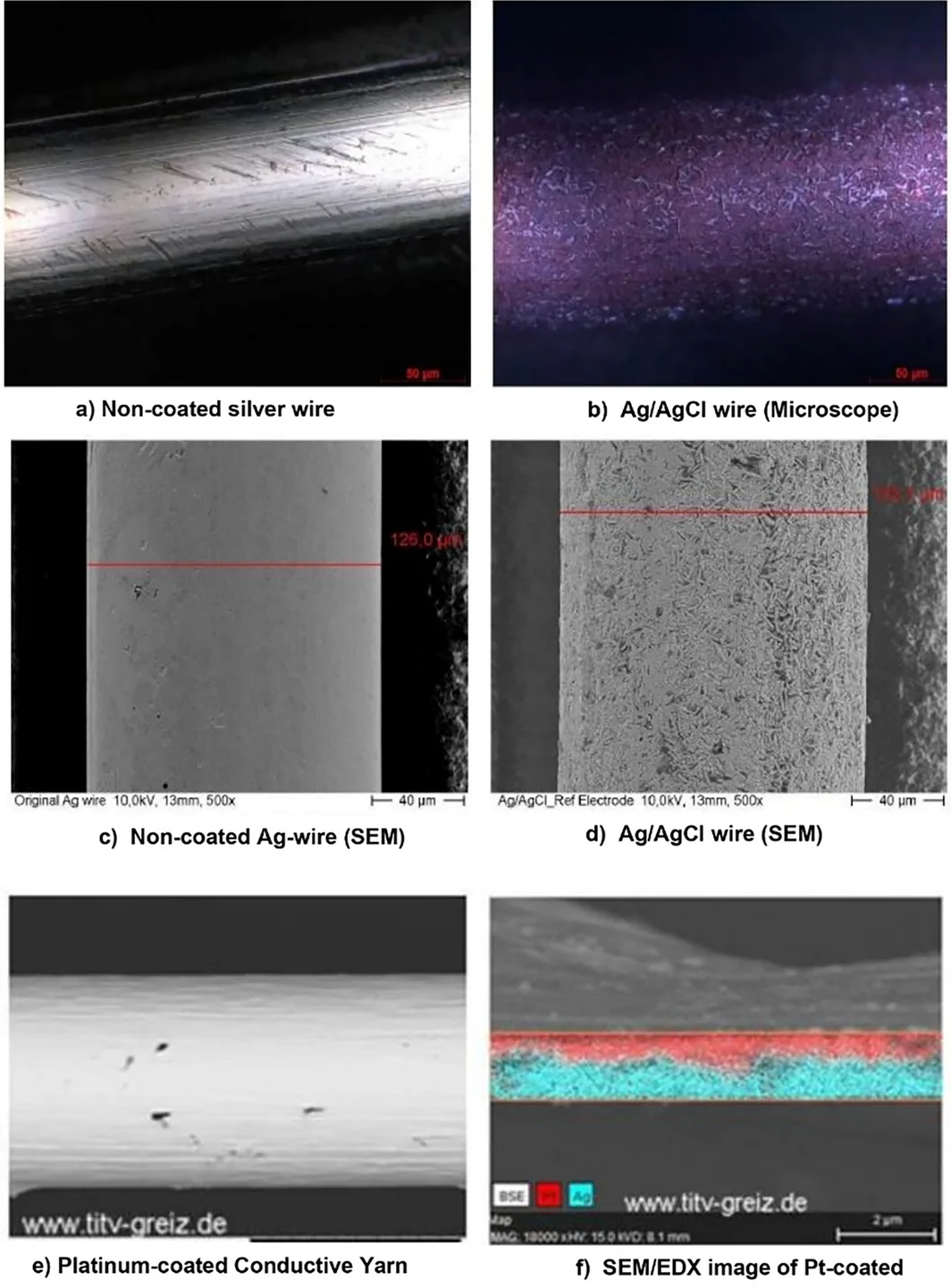
Microscopic and SEM views of sensor electrodes: (a) optical image of uncoated silver wire; (b) optical image of Ag/AgCl‐coated silver wire; (c) SEM image of uncoated silver wire; (d) SEM image of Ag/AgCl‐coated silver wire; (e) SEM image of platinum‐coated Shieldex conductive yarn; (f) SEM/EDX elemental map of platinum‐coated Shieldex conductive yarn showing platinum (red) over silver (blue).

For the working electrode in the redox sensor, SEM and EDX imaging indicated the presence of a continuous platinum layer on the silver‐plated Shieldex polyamide monofilament. Figure 9e,f shows representative images of the Pt‐coated conductive yarn: panel (e) illustrates a generally uniform surface morphology, while panel (f) presents the EDX elemental map highlighting the platinum‐rich outer layer over the silver substrate. No pronounced coating discontinuities or delamination were observed within the examined regions. These structural observations support the suitability of the fabricated electrodes for textile‐integrated electrochemical sensing and align with the electrochemical response behaviour reported in the redox experiments.

### 3.4 Cytocompatibility of Lactate and Redox Sensor Materials

The cytocompatibility of selected electrode materials was evaluated by LDH activity using NHDF fibroblasts in extract‐based testing, following incubation periods of 1, 7, 14 and 21 days. Figure 10 shows the cell numbers after exposure to extracts of PA/AgPt, Au‐PPD‐Chi‐GA‐LOx, Ag/AgCl, Pt and the reference control. Overall, the tested materials supported cell proliferation throughout the observation period. Gold‐ and platinum‐based electrodes exhibited cell numbers comparable to the reference at all time points, indicating good cytocompatibility under the tested in vitro conditions. The PA/AgPt redox electrode also showed proliferation values close to the control, suggesting acceptable material tolerance in extract‐based exposure.

**FIGURE 10:**
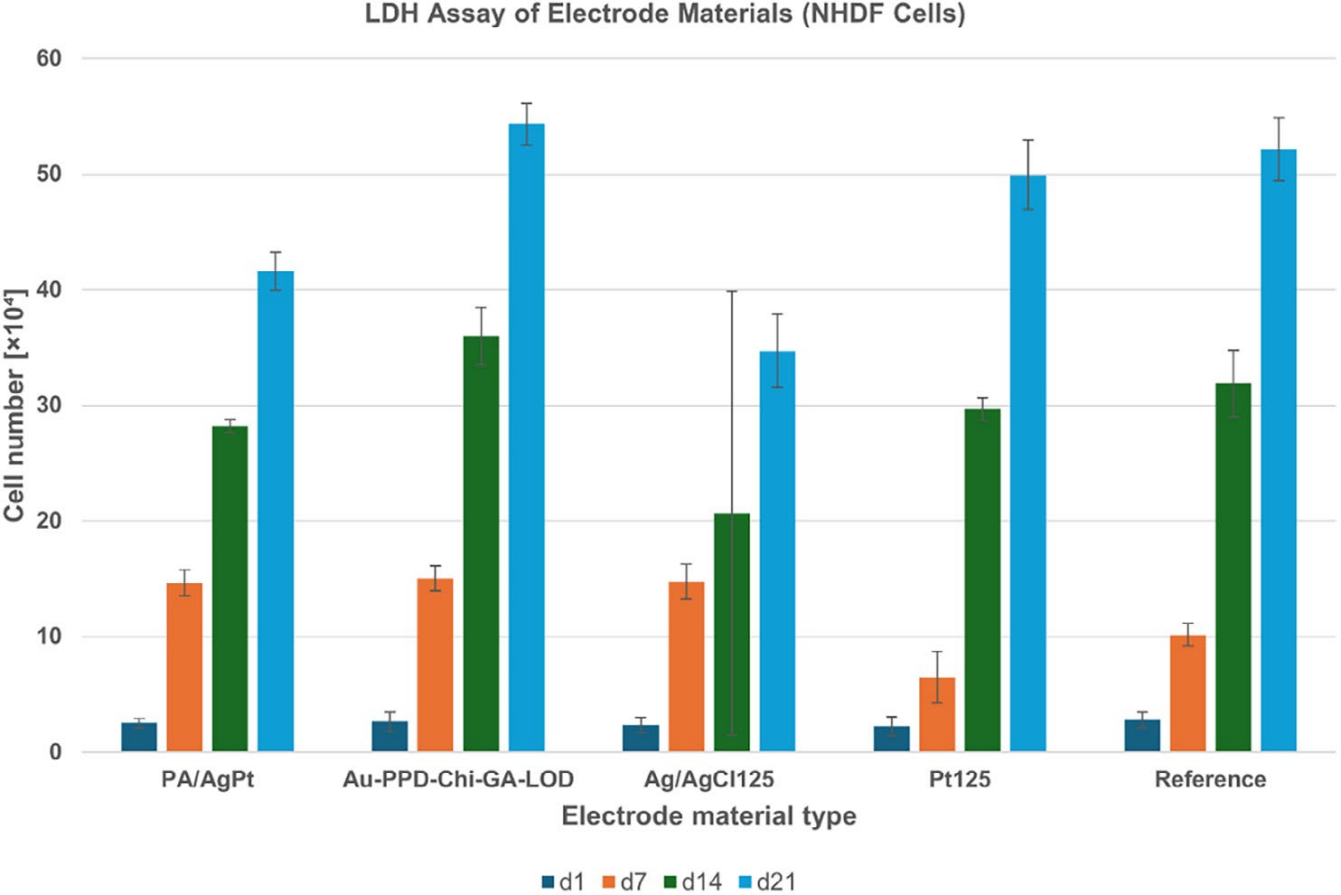
Cytocompatibility of electrode materials assessed by LDH activity in NHDF fibroblasts after extract‐based exposure for 1, 7, 14 and 21 days.

For Ag/AgCl, reduced proliferation trends were observed at prolonged incubation times (Days 14 and 21). The relatively large error bars on Day 21 indicate variability among replicates, suggesting that the observed effect was not uniform across samples. This variability may reflect concentration‐ or time‐dependent effects of silver‐containing materials, which have been reported previously in wound‐contact applications. Although this observation highlights the need for further investigation, the results suggest that silver‐based electrodes may exhibit time‐dependent biological responses depending on material configuration and exposure duration. Gold‐ and platinum‐based electrodes demonstrated favourable cytocompatibility in extract‐based testing, while silver‐containing electrodes should be interpreted with caution for prolonged exposure scenarios, particularly in wound‐contact environments.

## 4 Discussion

### 4.1 Redox Sensor

The winding‐structured textile redox sensor developed in this study, using Pt‐coated Shieldex monofilament working electrodes and Ag/AgCl reference electrodes, exhibited measurable and repeatable amperometric current–time response behaviour across a broad experimentally explored hydrogen peroxide concentration range (from the micromolar to high millimolar range) in PBS (pH 7.4), with signal equilibration occurring on the order of ~180 s. These characteristics are comparable to textile‐based ion‐sensing yarns produced via sheath–core configurations conducted by Ma et al. [11], which also emphasise uniform coating, mechanical robustness, and functional stability under repeated deformation. In contrast to flexible H_2_O_2_ sensors on paper or polymer substrates that achieve ultra‐low detection limits (100 nM–1 mM) but may lack robustness in moist environments [23], the present low‐potential (+12 mV vs. Ag/AgCl) textile winding design prioritises tolerance to protein‐rich wound exudate and robust operation in textile‐integrated configurations. This trade‐off aligns with other textile‐native sensing platforms, such as multi‐thread PEDOT:PSS‐based designs for pH/Cl^−^ detection reported by Possanzini et al. [22], which also demonstrate durable performance when embedded into wearable garments. Differences in substrate choice (Pt‐coated textile vs. carbonaceous or paper‐based), hydrodynamic environment and sample volume contribute to variations in detection limit and response time, but the current design is intended primarily for application‐oriented feasibility assessment and textile integration, rather than extreme laboratory‐based analytical performance. Accordingly, the present sensor architecture emphasises pre‐clinical feasibility and integration into textile‐based wound dressing concepts, while comprehensive analytical optimisation remains the subject of future work. This distinction underscores that the sensor's primary value lies in durable textile integration and wound‐application relevance, rather than benchmark detection limits under idealised laboratory conditions.

The principal contribution of the present work lies not only in the electrochemical response behaviour of the sensing elements but also in the development of textile‐compatible sensor architectures that facilitate practical integration into wound dressing materials. Compared with many previously reported textile electrochemical biosensors, the combination of a coaxial winding redox sensor, a triple‐helix braided enzymatic lactate sensor and textile‐compatible fabrication strategies provides an application‐oriented sensing platform for future smart wound dressing systems. This architecture‐driven approach emphasises mechanical flexibility, textile process compatibility and practical integration into wound‐care textiles while maintaining electrochemical functionality for wound‐relevant biomarker assessment.

### 4.2 Lactate Sensor

The lactate sensing platform developed in this work employed a gold working electrode functionalized with a PPD–chitosan–LOx multilayer, enabling enzymatic lactate‐responsive behaviour through conversion to hydrogen peroxide. Current–time measurements at constant potential demonstrated consistent response behaviour at physiologically relevant concentrations (2–4 mM), corresponding to lactate levels observed in exudates from chronic and healing wounds. Environmental sensitivity tests showed measurable baseline shifts under pH (6.0–8.0) and temperature (25°C–40°C) variation, which is in line with previous reports on textile‐based lactate biosensors, especially by Soekoco et al. [16], where environmental conditions influenced sensor output due to changes in enzyme activity and membrane permeability. The use of a permselective PPD membrane in combination with a hydrogel‐based chitosan layer likely contributed to reduced response contributions from larger electroactive species, similar to previously reported LOx‐based architectures [17].

Electropolymerized poly(phenylenediamine) (PPD) membranes have previously been shown to improve the selectivity of enzyme‐based electrochemical biosensors by limiting the transport of common electroactive interfering species while maintaining permeability towards hydrogen peroxide [25, 26]. In the present study, the PPD membrane was incorporated following these established design principles to improve the selectivity of the lactate sensing platform. However, systematic interference studies using physiologically relevant wound‐associated electroactive species and biomolecules were beyond the scope of this proof‐of‐concept investigation. Comprehensive selectivity evaluation will therefore form an important part of future analytical validation.

However, in line with observations from [17], repeated‐use tests showed gradual signal attenuation, attributable in part to lactate oxidase leaching and reduced hydrogel adhesion to the electrode surface. When compared with recent enzymatic electrochemical platforms incorporating environmental compensation mechanisms [24], the current design offers the advantage of a fully textile‐integrated electrode arrangement in a triple‐helix braid, which supports mechanical flexibility and integration into wound dressings. The absence of active pH or temperature correction mechanisms means that absolute accuracy under uncontrolled conditions may be reduced, a limitation also reported for other textile‐integrated enzymatic biosensors.

Similar environmental influences on enzymatic electrochemical biosensors have been reported previously, where optimised permselective membranes and signal‐processing strategies have been employed to compensate for variations in pH and temperature [17, 24]. In the present study, no active compensation strategies were implemented, as the primary objective was to evaluate the intrinsic response behaviour of the textile‐integrated sensing platform under wound‐relevant environmental conditions. Future developments may combine improved enzyme stabilisation, optimised permselective coatings and electronic compensation algorithms to further enhance sensor performance under dynamic wound environments.

Nevertheless, the observed response behaviour demonstrates the feasibility of integrating an enzymatic lactate sensing platform into a textile‐compatible braided architecture suitable for future wound dressing applications. Together, the combination of mechanical robustness from the textile‐engineered configuration and biochemical functionality from the multilayer enzyme system shows the potential of this approach for future wound‐sensing applications.

### 4.3 Cytotoxicity and Biocompatibility

Our LDH‐based cytocompatibility testing (Figure 10) indicates that gold‐ and platinum‐based electrodes maintain cell proliferation comparable to the reference control throughout 21 days, while Ag/AgCl shows a moderate reduction in proliferation at prolonged incubation times (Days 14 and 21). The large error bars observed at Day 21 for Ag/AgCl suggest variability among replicates, indicating that while the effect is noticeable, it is not uniformly pronounced.

These results are consistent with earlier studies on the biocompatibility of electrode materials. Our findings regarding gold electrodes align with the study by Demurtas et al., which reported minimal cytotoxicity and favourable in vivo performance of nanoporous gold electrodes for glucose sensing [27]. Similar to our observation that gold‐based lactate sensor electrodes demonstrated good tolerance in NHDF fibroblast cultures [28], Zamani et al. emphasised the extensive use of gold in biosensing applications because of its inertness and lack of cytotoxicity. Our observation of favourable proliferation and cytocompatibility in platinum electrodes aligns with Geninatti et al., who documented minimal cytotoxicity of Pt electrodes in fibroblast cultures, supporting widespread use of platinum as a biocompatible and conductive electrode material [29]. In contrast, Ag/AgCl electrodes exhibited moderate reductions in cell proliferation at later time points, consistent with reports on time‐ and concentration‐dependent effects of silver ion release in wound‐related applications [30].

Taken together, these results support the suitability of gold and platinum electrodes as primary sensing elements for wound‐contact biosensors, while highlighting the need for careful consideration of silver‐based components in long‐term applications. Although Ag/AgCl remains a widely used and effective reference electrode material for electrochemical sensing, future textile sensor designs may benefit from protective encapsulation strategies or optimised electrode configurations to minimise prolonged tissue exposure and potential silver ion release when extended wound contact is anticipated. These findings further support the feasibility of integrating gold‐ and platinum‐based textile electrodes into future smart wound dressing systems while emphasising the importance of material selection for long‐term clinical applications.

### 4.4 Overall Assessment and Study Limitations

The present study was designed as a proof‐of‐concept investigation to demonstrate the feasibility of textile‐integrated electrochemical sensing for wound‐relevant biomarkers. Accordingly, the electrochemical evaluation focused on qualitative response behaviour under controlled laboratory conditions rather than comprehensive analytical validation.

Parameters such as analytical sensitivity, detection limits, selectivity towards interfering wound constituents, long‐term operational stability, calibration performance and interference testing require systematic investigation in future studies. Although the lactate sensor incorporated a permselective poly(phenylenediamine) (PPD) membrane based on established biosensor design principles, comprehensive interference studies with physiologically relevant wound‐associated electroactive species and biomolecules were beyond the scope of the present investigation and will form an important part of future analytical validation.

PBS was selected as a standard supporting electrolyte to establish the baseline electrochemical behaviour of the textile‐integrated sensors while minimising biological variability. Using a chemically well‐defined buffer allows the intrinsic electrochemical responses of the sensor architectures and electrode materials to be characterised under controlled conditions, without the additional complexity introduced by proteins, enzymes, cells and variable ionic compositions present in wound exudates [31, 32]. Such controlled characterisation is commonly adopted during the early stages of electrochemical biosensor development before subsequent evaluation in increasingly complex biological media [33]. The use of PBS in the present study is therefore consistent with the proof‐of‐concept objective of establishing the fundamental electrochemical performance of the proposed textile sensing platform prior to evaluation under biologically relevant wound conditions. Evaluation in simulated wound fluid and authentic wound exudate therefore represents an important next step towards analytical and translational validation and will be the focus of future work.

Similarly, the mechanical evaluation was limited to preliminary bending‐cycle experiments that demonstrated retention of sensor functionality following repeated deformation. More extensive durability studies, including higher‐cycle deformation testing, repeated washing, prolonged continuous operation and long‐term storage stability, were beyond the scope of the present investigation and will be important for future translational development.

Overall, the present work demonstrates the feasibility of architecture‐driven textile electrochemical sensing using textile‐compatible fabrication strategies for wound‐related biomarker monitoring. While further analytical validation, comprehensive selectivity studies, extended mechanical durability evaluation and assessment under biologically relevant wound conditions remain necessary before clinical translation, the proposed textile sensor architectures provide a promising foundation for the future development of smart wound dressing systems.

### 4.5 Translational Perspective

From a translational perspective, the textile‐compatible sensor architectures developed in this study are intended for integration into flexible wound dressing systems capable of continuous monitoring of wound‐related biochemical changes. The proposed dual‐analyte textile sensing platform combines electrochemical functionality with textile‐compatible fabrication, providing a practical basis for future smart wound dressing systems. The yarn‐based construction facilitates incorporation into textile substrates using established textile manufacturing technologies such as embroidery or other textile integration techniques while maintaining the flexibility and conformability required for wound‐contact applications. In future wearable systems, the textile sensors may be combined with miniaturised electronics for signal acquisition, wireless data transmission and continuous patient monitoring. Integration with portable data acquisition systems may also facilitate real‐time assessment of wound status and support clinical decision‐making during wound management. Although long‐term operational stability, packaging strategies, sterilisation compatibility under repeated use, and clinical validation remain to be comprehensively investigated, the present work establishes a foundation for the future development of smart wound dressings capable of supporting objective monitoring of wound‐related biochemical changes in both clinical and home‐care environments.

## 5 Conclusion

This study presents an architecture‐driven, dual‐mode textile‐integrated electrochemical sensing platform for wound‐relevant biomarkers, specifically hydrogen peroxide (H_2_O_2_) and lactate. Using platinum‐coated Shieldex working electrodes and Ag/AgCl reference electrodes in a coaxial winding structure yarn, the redox sensor was successfully integrated into a mechanically stable textile substrate and functionally evaluated under physiologically relevant conditions. For lactate sensing, a gold‐based working electrode was functionalized with a chitosan–PPD–LOx enzymatic layer in a three‐electrode setup with an Ag/AgCl reference and platinum counter electrode. Both sensors exhibited consistent qualitative current–time response behaviour towards their respective analytes, demonstrating the feasibility of architecture‐driven textile integration for wound‐relevant biochemical sensing.

The redox sensor showed a measurable response to hydrogen peroxide across a broad experimentally explored concentration range, with signal evolution approaching equilibrium after approximately 180 s. Environmental factors, such as pH and temperature, caused baseline shifts that need to be taken into account for practical use. However, the results demonstrate a robust qualitative response trend within hydrogen peroxide concentration ranges relevant to wound environments.

The lactate sensor showed enzyme activity over multiple measurement cycles and responded to physiologically relevant lactate concentrations. The use of a permselective PPD membrane and a hydrogel‐based enzyme matrix supported enzymatic response behaviour, although variations in pH and temperature influenced signal output. While the platform achieved measurable responses within the wound‐relevant lactate range of exudates, the operational stability of hydrogel adhesion and enzyme immobilisation over extended use remains an important consideration for ensuring reliable performance.

Evaluation of cytocompatibility indicated that electrodes made of gold and platinum supported normal fibroblast growth comparable to that of reference controls. These findings suggest that gold and platinum electrodes are suitable candidates for wound‐contact sensing applications, while highlighting the need for careful material selection and design considerations when silver‐based components are used under prolonged exposure conditions.

Future research should concentrate on improving enzyme immobilisation strategies, integrating temperature and pH compensation mechanisms, performing comprehensive analytical validation (including calibration, detection limits, selectivity, interference testing and long‐term stability), evaluating sensor performance in simulated wound fluids and authentic wound exudates, conducting comprehensive mechanical durability and long‐term operational reliability studies under repeated textile deformation and further developing sensor packaging, wearable electronic integration for wireless data acquisition and clinical validation to support translational implementation. The dual‐analyte textile‐based sensing approach described here demonstrates application‐oriented feasibility for integration into smart wound dressings aimed at supporting qualitative monitoring of biochemical changes in wound environments for future clinical and home‐care‐oriented applications.

## Funding

This work was supported by funds of the Federal Ministry for Economic Affairs and Energy on the basis of a decision passed by the German Parliament.

## Ethics Statement

This study did not involve human participants.

## Conflicts of Interest

The authors declare no conflicts of interest.

## Data Availability

The data that support the findings of this study are available from the corresponding author upon reasonable request.
